# Analysis of Postprandial Glycemia in Relation to Metabolic Compensation and Other Observed Parameters of Outpatients with Type 2 Diabetes Mellitus in the Czech Republic

**DOI:** 10.1007/s13300-018-0379-3

**Published:** 2018-02-19

**Authors:** Denisa Janíčková Žďárská, Martin Hill, Milan Kvapil, Pavlína Piťhová, Jan Brož

**Affiliations:** 1 Department of Internal Medicine, Second Faculty of Medicine, Charles University, Prague, Czech Republic; 2 Institute of Endocrinology, Prague, Czech Republic

**Keywords:** Diabetes mellitus, Fasting glycemia, Glycosylated hemoglobin, Metabolic compensation, Postprandial glycemia

## Abstract

**Introduction:**

The goal of the study was to determine the level of metabolic compensation expressed by glycosylated hemoglobin, fasting plasma glucose, and postprandial glucose as determined after a standardized breakfast; further, to evaluate interrelationships between the studied parameters and postprandial glucose levels.

**Methods:**

The study included 1055 patients with type 2 diabetes mellitus. Their fasting plasma glucose and postprandial glucose were measured before and after a standardized breakfast. Attending diabetologists completed a uniform questionnaire that included demographic data, type of antidiabetic treatment, duration of diabetes, latest glycosylated hemoglobin value, presence of dyslipidemia, and organic complications.

**Results:**

Glycosylated hemoglobin < 53 mmol/mol was achieved in 363 (34.2%), postprandial glucose < 7.5 mmol/l in 211 (19.9%), and fasting plasma glucose < 6 mmol/l in 251 (23.7%) patients. Excellent metabolic compensation, indicated by all the above mentioned glycosylated hemoglobin, fasting plasma glucose, and postprandial glucose values simultaneously, was achieved in only 71 (6.7%) patients. Comparable to fasting plasma glucose and postprandial glucose values, correlation with glycosylated hemoglobin levels is statistically significant; however, there is no difference at different glycosylated hemoglobin levels. There was a significant correlation between dyslipidemia and postprandial glycemia (*p* = 0.013).

**Conclusion:**

The objective of care for patients with diabetes mellitus is to improve their long-term metabolic compensation; to that end, both fasting plasma glucose and postprandial glucose deserve equal attention.

## Introduction

DM (diabetes mellitus) is a progressive disease which over the years leads to metabolic complications [1, 2]. Formerly, the risk of these complications has been associated only with the level of glycosylated hemoglobin (HbA_1c_) which reflects long-term changes in glucose metabolism. At present, there are data available that indicate a close relationship between the development of late complications and postprandial glycemia (PPG), which is an independent risk factor of organic complications [3–6]. Taken together, PPG, fasting plasma glucose (FPG), and HbA_1c_ then serve to assess metabolic compensation in the wider sense, forming the so-called glucose triad, all components of which should be addressed by treatment [7, 8]. Target values of PPG, FPG, and HbA_1c_ should be individualized, particularly with regard to the choice of antidiabetic medication (risk of hypoglycemia), DM duration, life expectancy, presence of concurrent disorders, and complications [9].

### Postprandial Plasma Glucose

Postprandial plasma glucose levels are a direct measure of glucose concentrations in the blood following a meal, standardized generally at 2 h after eating (2 h PPG). In healthy individuals, glucose levels reach a peak approximately 1 h after ingestion of food and then return to premeal levels within 2–3 h [10]. Normal 2 h PPG levels are usually < 6.6 mmol/l and should not be > 7.8 mmol/l [11]. Such targets are individualized particularly with regard to the age of each patient and associated organic complications.

Postprandial hyperglycemia is a frequent occurrence in patients with type 2 diabetes, even at normal HbA_1c_ levels, when PPG may become elevated. In a number of studies with type 2 DM patients that recorded glycemic profiles including PPG, elevation of PPG up to 8.9 mmol/l has been noted, despite HbA_1c_ below 54 mmol/mol [12, 13].

### PPG and its Relationship to Cardiovascular Disease

The relationship between hyperglycemia and cardiovascular disease is complex, with evidence suggesting that an acute increase of glycemia, particularly after a meal, may have a direct detrimental effect on cardiovascular disease [14–17]. The value of PPG monitoring has been demonstrated by analysis of the Diabetes Epidemiology: Collaborative Analysis of Diagnostic Criteria in Europe (DECODE) study population. The DECODE study group reported that 2 h PPG levels are a better predictor of on-study death from all causes and from cardiovascular disease than FPG levels [3].

Evidence from more recent studies notes that a marker of atherosclerosis corresponds better with the peak magnitude of patients’ postprandial glucose excursions than with both FPG and HbA_1c_ levels [18, 19]. Specifically, it is thanks to knowing the FPG and PPG values that we are able to determine the extent of the variability which is the risk factor of organ damage. This fact was demonstrated also with relatively new technologies, such as the sensors of continuous glucose monitoring [20]. A study by Buscemi et al. [20] suggests that glycemic variability influences endothelial function even in non-diabetic subjects. Such variability may explain the increased cardiovascular risk observed in patients prior to developing overt type 2 DM. The negative impact of glycemic variability on vascular endothelium function can be explained by many factors, mainly by hyperglycemic memory with activation of oxidative stress [21], even in healthy individuals [22].

### Primary Objective

The goal of epidemiological analysis of diabetic outpatients in the Czech Republic was to determine the level of metabolic compensation expressed by HbA_1c_, FPG, and PPG, and to determine the percentage of patients meeting the excellent metabolic compensation parameters according to standards of the Czech Diabetes Society.

### Secondary Objective

To investigate the relationships between the collected demographic factors (gender, age, BMI, dyslipidemia, persistence length of diabetes, presence of late complications of diabetes, type of antidiabetic treatment, HbA_1c_, FPG) and measured level of PPG.

To determine the contribution of the FPG and PPG to different levels of HbA_1c_.

To evaluate the relevance of measured PPG levels for potential treatment change in subpopulations of diabetic patients.

## Methods

This was an observational multicenter study with participation of physicians from diabetes outpatient departments in the Czech Republic. In total, the study involved 1055 subjects with type 2 DM. Recruited outpatients came from consulting rooms of general diabetologists and were included as they attended ordinary visits to their doctor. The only inclusion criteria for the study were type 2 DM, age > 18 years, and signed informed consent. The level of HbA_1c_ (values are given in millimoles per mole according to the IFCC calibration method) was not an exclusion/inclusion criterion in the study. The patients’ FPG and PPG were measured before and after a standardized breakfast—a ham baguette Crocodile (contains 268.4 kcal, 11.52 g protein, 22.70 g carbohydrates, 16.56 g lipids). Attending diabetologists completed a uniform questionnaire that included demographic data, type of antidiabetic treatment, DM duration, latest know HbA_1c_ value, presence of dyslipidemia and organic complications, and finally a response to a query concerning the significance of PPG for further treatment. Metabolic compensation target values were assessed by current care standards of the Czech Diabetes Society (http://www.diab.cz) [23].

The relationships between compensation indicators were evaluated by 2-factor ANOVA for interaction between the factors. The ANOVA was followed by multiple comparison tests (least significant difference test). Simultaneous evaluation of the relationships between indicators of diabetes compensation and the studied parameters was carried out by multiple regression with dimension reduction. Dichotomic data dependencies were tested by Fisher’s exact test. Levels of PPG in subjects with dyslipidemia vs subjects with normal serum lipids were evaluated using robust Mann–Whitney tests.

All procedures followed were in accordance with the ethical standards of the responsible committee on human experimentation (institutional and national) and with the Helsinki Declaration of 1964 (as revised in 2013). Informed consent was obtained from all patients to be included in the study.

## Results

In total, 1055 subjects with type 2 DM participated in the study. The characteristics of the patients are presented in Table 1.

**Table 1:** Characteristics of patients

	*n*	HbA_1c_ (mmol/mol)	PPG (mmol/l)	FPG (mmol/l)	BMI (kg/m^2^)	Duration of DM (years)	Age (years)
OADs	732	55.55 (55.11; 55.99)	9.51 (9.41; 9.62)	7.01 (6.94; 7.07)	30.52 (30.36; 30.68)	8.64 (8.44; 8.85)	66.74 (66,41; 67.08)
Insulin therapy with/without OAD	292	71.31 (70.31; 72.32)	11.53 (11.31; 11.75)	8.39 (8.25; 8.53)	30.42 (30.16; 30.69)	12.62 (12.20; 13.10)	63.91 (63.54; 64.26)

Data is presented as mean (lower; upper interval)

*OADs* oral antidiabetic drugs, *HbA*_*1c*_ glycosylated hemoglobin, *PPG* postprandial glycemia, *FPG* fasting glycemia, *BMI* body mass index

Within the framework of metabolic compensation evaluation, HbA_1c_ < 53 mmol/mol was achieved in 363 (34.2%), PPG < 7.5 mmol/l in 211 (19.9%), and FPG < 6 mmol/l in 251 (23.7%) patients. Excellent metabolic compensation, indicated by all the above HbA_1c_, FPG, and PPG values simultaneously, occurred in only 71 (6.7%) patients.

In our study PPG correlated with HbA_1c_ levels comparably with FPG. The correlation for FPG was *r* = 0.472 (*p* < 0.001) and for PPG it was *r* = 0.491 (*p* < 0.001). The correlation for PPG vs FPG did not statistically differ. The correlation of PPG of the group on oral antidiabetic agents (OADs) only vs those on insulin with/without OADs was *r* = 0.433 (*p* < 0.001) vs *r* = 0.371 (*p* < 0.001) and for FPG it was *r* = 0.384 (*p* < 0.001) vs *r* = 0.388 (*p* < 0.001). The impact of antidiabetic medication on the level of PPG was not found.

In the studied group the PPG contribution did not differ at different HbA_1c_ levels.

Patients with dyslipidemia had increased PPG levels vs patients with normal lipid control—9.4 mmol/l (7.8; 12.0) vs 10.1 mmol/l (8.0; 12.8) [median (lower; upper limit)]. The difference was statistically significant (*p* = 0.013).

Microvascular complications were present in 350 (33.2%) and macrovascular complications in 355 (33.6%) patients.

According to the responding doctors, PPG was relevant for change in treatment in 807 cases (76.5%).

Antidiabetic treatment of study subjects is presented in Table 2.

**Table 2:** Treatment of patients with diabetes mellitus recruited into the study

	*n*	%
Sulfonylurea derivatives	391	36.9
Metformin	587	55.4
Glitazones	46	4.34
Other oral antidiabetic drugs	37	3.49
Insulin	305	28.9
Premixed human insulins	47	4.43
Premixed insulin analogues	30	2.83
Basal insulin (human/analogues)	47/18	4.43/1.7
Intensive insulin regimens (human)	96	9.06
Intensive insulin regimens (analogues)	32	3.02
Combination of human insulins + analogues	28	2.64
Prandial insulin only	7	0.66

## Discussion

The study was designed to determine the level of metabolic compensation, particularly the level of PPG in the diabetic outpatient population in the Czech Republic. Its aim was not to test individual treatment regimens but rather the parameters of glucose metabolism compensation and under different treatments. HbA_1c_ < 53 mmol/mol was achieved in 363 (34.2%), PPG < 7.5 mmol/l in 211 (19.9%), and FPG < 6 mmol/l in 251 (23.7%) patients. Excellent metabolic compensation, indicated by achieving these HbA_1c_, PPG, and FPG values simultaneously, as recommended by the Czech Diabetes Society, was achieved in only 71 (6.7%) patients.

A discrepancy in the percentage representation of individual parameters of the target metabolic compensation must be pointed out. If only 6.7% of the patients meet all three parameters, we can estimate that, for example, a PPG < 7.5 mmol/l will not bring about a target HbA_1c_ < 53 mmol/mol, in which case the desired PPG value could be higher, such as < 10.0 mmol/l.

PPG measurement is still often neglected, particularly in type 2 DM patients, who do not receive insulin treatment and in whom frequently only FPG is being determined. And yet, a DM patient is in a postprandial state for most of the time during the day and PPG therefore can markedly affect the resulting HbA_1c_. Recent studies have focused on determining its PPG contribution to overall HbA_1c_ levels. Reports indicate that postprandial hyperglycemia contributes approximately 70% of the total glycemic burden at HbA_1c_ levels < 56 mmol/mol, decreasing to around 30% at HbA_1c_ levels > 89 mmol/mol. The contribution of PPG to the resulting HbA_1c_ is greater, the lower the HbA_1c_ is [24, 25]. In contrast, the contribution of FPG increases with increasing HbA_1c_ levels, suggesting that PPG may be a better indicator of glycemic control than FPG in patients with moderately elevated blood glucose [26]. Support for this hypothesis is provided by data suggesting that treatment aimed at reducing postprandial glucose excursions is more effective in lowering HbA_1c_ levels than FPG-targeted therapy [27]. A number of randomized controlled trials have shown that patients treated with twice daily biphasic insulin, incorporating a rapid-acting analogue, achieved significantly lower HbA_1c_ levels, compared with patients receiving a long-acting basal insulin [28–30]. Antidiabetic medication preferentially targeting PPG levels can bring other benefits as well, such as alleviation of endothelial dysfunction. Regimens using rapid-acting insulin analogues are effective both in reducing arterial oxidative stress and in improving endothelial dysfunction [31, 32]. OADs from the α-glucosidase inhibitors (AGIs), glinide classes, and gliptins have also been shown to improve markers of atherosclerosis in patients with type 2 diabetes [33–35]. Indeed, the benefits of the AGI acarbose translate into significant reduction in the risk of cardiovascular disease in patients with prediabetes, impaired glucose tolerance [36]. As the PPG is an independent risk factor of vessel wall damage it should be considered in the comprehensive management plan of individuals with diabetes. This should be taken into account when choosing antidiabetic medication, which should primarily target PPG [27].

We tested the impact of antidiabetic medication of participating subjects on their levels of PPG. This hypothesis was not borne out by our study. None of the administrated medications had any favorable impact on PPG levels. The group treated only with OADs did not differ in the impact on PPG compared to insulin (with/without OADs) treated patients.

In our study we investigated the contribution of PPG and FPG to overall HbA_1c_ levels. There are insufficient data to determine accurately the relative contribution of the FPG and PPG to HbA_1c_ It appears that FPG is somewhat better than PPG in predicting HbA_1c_, especially in type 2 diabetes [10]. In our study PPG correlated with HbA_1c_ levels comparably with FPG. In the studied group, the PPG contribution did not differ at various HbA_1c_ levels. This can be explained by the fact that the range of studied HbA_1c_ values were below threshold for suboptimal control, especially in those patients on OADs. (The level of HbA_1c_ was not an exclusion/inclusion criterion in the study.) We also did not find a different contribution of the PPG level in patients treated with insulinotherapy where the HbA_1c_ was higher [for OADs 55.55 mmol/mol (55.11; 55.99) vs insulinotherapy 71.31 mmol/mol (70.31; 72.32)].

In our study we found increased PPG level in patients with dyslipidemia vs patients with normal lipid control (*p* = 0.013). Hyperlipidemia and hyperglycemia together represent a malignant combination for a risk of vascular complication. It is generally understood that dyslipidemia is closely related to metabolic compensation mainly in case of type 1 DM patients, whilst in case of patients with type 2 DM the lipid profile is more likely to be a factor of insulin resistance and metabolic syndrome. On the other hand our study showed that PPG correlates with dyslipidemia also in the case of type 2 DM patients. Chronic elevations of glucose and/or lipids might damage β-cells, eventually enhancing pre-existing insulin resistance and insulin deficiency (glucolipotoxicity). Both abnormalities should be therefore addressed in the treatment strategy.

In our study only 55.4% of patients were treated with metformin. Even if metformin treatment contraindications (renal, respiratory, or cardiac insufficiency) are taken into account, the frequency of its administration can be considered deficient with regard to guidelines for type 2 DM treatment [9].

## Conclusions

The objective of care for DM patients is to improve their long-term metabolic compensation; to that end, FPG and PPG deserve equal attention, as both represent measures essential for prevention of cardiovascular disorders in diabetics.
